# Exosomes in Plastic Surgery: An Expert Video Roundtable Discussion

**DOI:** 10.1093/asjof/ojaf093

**Published:** 2025-07-12

**Authors:** Steven Cohen, Brannon Claytor, Diane Duncan

The authors are pleased to present an expert roundtable discussion on exosomes in plastic surgery (Video). The discussants include a panel of leading experts in aesthetic surgery, and the video roundtable was recorded live at The Aesthetic MEET 2025 in Austin, TX. The panelists discussed the history of exosome research and described the recent advancements that have led to the use of exosomes in wound healing, skin rejuvenation, and other aesthetic treatments. They discussed some of the challenges regarding efficacy, safety, and FDA approval for exosome products, and the complexities of determining dose and usage of exosome products. They then discussed their use of exosomes in their own practices and agreed that exosomes have shown promising results in treating ischemia and skin rejuvenation. They concluded by discussing the current regulations and process of FDA approval for exosome treatments and the many possibilities that lie ahead for exosomes in a wide variety of clinical uses.

